# Research on synchronous load control of shield beam for 50,000 kN hydraulic support test bench

**DOI:** 10.1371/journal.pone.0335435

**Published:** 2025-11-20

**Authors:** Chengfeng Wu, Tiangu Wu, Lijuan Zhao, Wenliang Li, Qingliang Zhang, Lin Li, Qing Chen

**Affiliations:** 1 Shandong Yankuang Intelligent Manufacturing Co., LTD, Zoucheng, Shandong, China; 2 School of Mechanical Engineering, Liaoning Technical University, Fuxin, Liaoning, China; Vellore Institute of Technology, INDIA

## Abstract

This paper proposes an innovative synchronous load control system for protective beams, addressing the issues of uneven loading and asynchronous positioning in dual-cylinder loading systems during hydraulic support shield beam loading experiments. The system integrates multi-domain modeling and co-simulation technologies using tools such as Simulink, AMESim, and Adams, creating a unified model encompassing mechanical, hydraulic, and control aspects. This approach enables precise perception, signal processing, and adaptive regulation of the dynamic loading process.The core of this research lies in the application of an adaptive RBF-PID control strategy, benchmarked against a fuzzy PID controller. Simulation results demonstrate that the RBF-PID controller exhibits significant advantages in handling uneven loads and achieving rapid resynchronization, with a 61% reduction in maximum synchronization error and a 34% improvement in resynchronization speed compared to the fuzzy PID control. Concurrently, stress analysis identified the pinhole connection position as the area with the most concentrated stress on the protective beam loading block, providing critical data support for structural strength design.Finally, experimental verification conducted on a 50000kN hydraulic support test bench validates the effectiveness and feasibility of this control strategy in real-world conditions. The experimental results are highly consistent with simulation outcomes, effectively resolving engineering challenges encountered during the protective beam loading process and offering new insights and methods for the optimization and control strategy development of hydraulic support test benches.

## 1 Introduction

As coal mining continues to deepen, the working environment of hydraulic supports is becoming increasingly harsh, with the requirements for load-bearing capacity, safety performance, and stability also constantly increasing. In this context, the study of hydraulic support test benches is of great practical significance. Hydraulic support test benches are crucial equipment for evaluating the safety and technical performance of hydraulic supports. Comprehensive and accurate testing of hydraulic supports can help discover potential problems in a timely manner, prevent safety accidents, and provide strong guarantees for mine safety production. Many universities, research institutions, and enterprises have invested in the research and development of hydraulic support test benches, achieving a series of results. These studies involve the design, manufacturing, optimization, and related control systems of hydraulic support test benches, addressing issues such as improving accuracy, reducing costs, and simplifying operations.

In research on synchronous control and strength of hydraulic support test benches, reference [1] analyzed the stress changes at various hinge positions of the shield beam under different impact loads applied at different positions, providing a reference for the stability and strength design of supports. Reference [2] analyzed the harsh working conditions of the shield beam when large mining height hydraulic supports are at low positions and proposed measures to prevent failure. Reference [3] studied the cracking phenomenon of the rear structural components of the shield beam during the compression process of large mining height supports and optimized the shield beam structure. Reference [4] established an adaptive cutting control strategy for coal mining machines through multi-domain modeling and collaborative simulation, verifying the correctness of this simulation method. Reference [5] used virtual prototype technology and deep convolutional neural network algorithms to build an adaptive height adjustment machine-fluid-control integrated system for coal mining machines, comparing it with fuzzy PID control algorithms to verify that deep convolutional neural network algorithms are more suitable for the control strategy of coal mining machine height adjustment systems. Reference [6] aimed to improve the precision of forging machine double-cylinder hydraulic synchronous control by using a single-neuron PID control algorithm and cross-coupling algorithm as the forging machine double-cylinder hydraulic synchronous control algorithm. Through simulation, the position tracking error, relative synchronous control error, and speed and pressure tracking error of the left and right hydraulic cylinders under the action of conventional fuzzy PID control algorithms and fuzzy-single neuron PID control algorithms were obtained and compared. The feasibility of the double-cylinder synchronous control method was verified through experiments. Reference [7] optimized the control performance of the proportional valve using a single-neuron PID control strategy and designed a dual-cylinder synchronous hydraulic control system with high synchronization accuracy using the optimized proportional valve. The system was studied and analyzed through simulation and related experiments. Reference [8] conducted an electro-hydraulic joint simulation of a four-cylinder synchronous control system for lifting mechanisms in Amesim/Simulink. Under a parallel fuzzy PID control strategy, the four-cylinder synchronous control system had high synchronization accuracy and small displacement tracking errors, further improving the performance of the lifting platform. Reference [9] implemented synchronous control through error feedback, achieving precise control of the synchronous operation process of double hydraulic cylinders. PID parameters were tuned using a genetic algorithm, significantly improving the synchronization control accuracy of the double-cylinder hydraulic system. Reference [10] proposed a BP neural network-based PID initial support force adaptive control method, established a three-layer neural network control model, and updated the weight coefficients of the output layer and hidden layer using supervised Hebb learning rules and gradient descent methods. The three control parameters of the PID controller were obtained through training. Reference [11] designed a nonlinear inverse control algorithm based on command filtering and neural networks, which effectively compensated for the effects of unmodeled dynamics and external disturbances on the electromechanical servo system, thereby improving the system’s control effects. Reference [12] proposed an RBF-PID control method suitable for vacuum circuit breaker motor actuation mechanisms, combined with a dynamic mathematical model of limited rotation angle permanent magnet brushless DC drive motors, analyzed the speed response characteristics and controllability of the motion process of drive motors.

**Fig 1:**
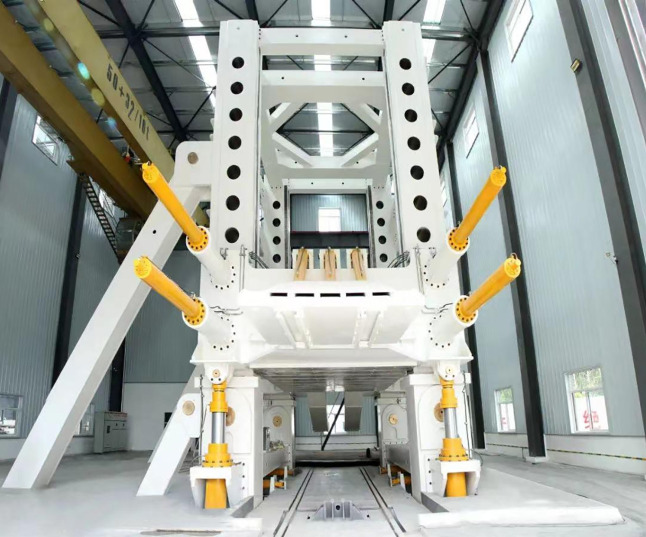
50000kN hydraulic support test bench.

Given the importance highlighted by the literature and research on the issues faced by high-cut hydraulic support canopy beams, various control strategies have been proposed to optimize system performance. Informed by the latest research, this study investigates the strength and control challenges during canopy beam loading experiments on our self-developed, state-of-the-art hydraulic support test platform, boasting a 50000 kN capacity and a 10-meter test height (Fig 1). This platform’s specific loading process and configuration present unique operational challenges, including significant external disturbances and induced oscillations. Utilizing a multi-domain modeling and simulation approach, coupled with neural network and fuzzy control techniques, we propose an RBF-PID adaptive control algorithm. Crucially, we have successfully utilized and tuned this RBF-PID controller to specifically address the external disturbances and oscillations arising from our platform’s unique loading conditions. Concurrently, stress distribution cloud maps provide data support for the strength design of loading blocks. The proposed method’s effectiveness is validated through simulation and experimentation. The key innovation lies in this tailored application of the RBF-PID control strategy, demonstrating its capability to effectively manage the complexities of our unique test platform and overcome the inherent synchronization and control issues, thereby providing effective engineering design solutions and novel approaches for advancing hydraulic support test platform development.

## 2 Model construction of cover beam loading system

### 2.1 Establishment of rigid flexible coupling model

**Fig 2:**
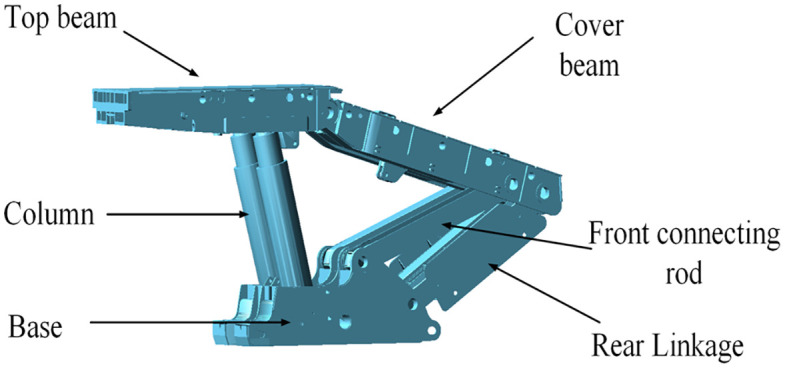
ZY29000/45/100d Hydraulic support model.

The ZY29000/45/100d hydraulic support is utilized as the experimental subject, with the model constructed in CERO and its height adjusted to 4.8m, as illustrated in Fig 2.

**Fig 3:**
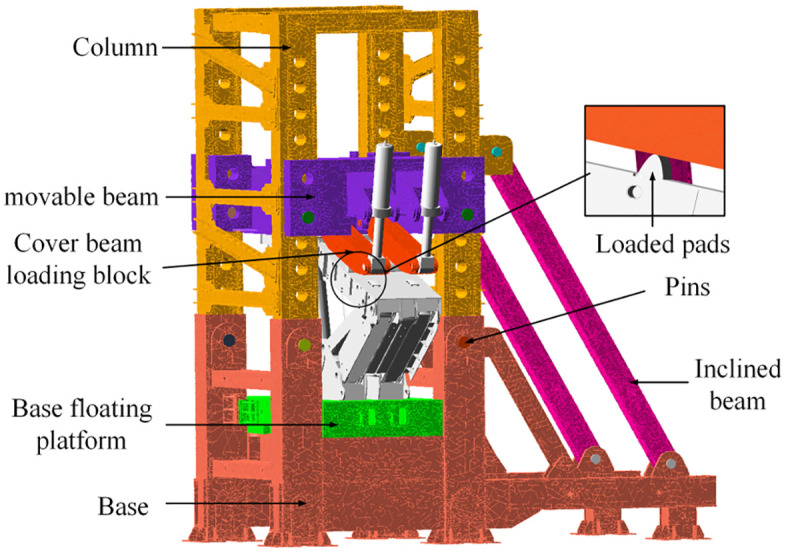
Assembly model of hydraulic support and test bench.

The hydraulic support test bench, developed by the project team, employs a 3D solid model created in CREO. Based on ANSYS, the test bench’s 3D solid model is converted into a flexible body and assembled with the hydraulic support’s 3D solid model shown in Fig 7. This results in a rigid-flexible coupling assembly model of the hydraulic support and test bench. Using the rigid-flexible coupling model for simulation not only enables the completion of dual-cylinder loading conditions but also facilitates the analysis of stress distribution in the loading device. This provides a data foundation for optimizing the structural design. The rigid-flexible coupling assembly model is depicted in Fig 3.

In the enlarged area of Fig 3, a loading pad with a width of 150 mm [13–15] and a length equal to the width of the shielding beam is added to ensure the even distribution of the loading force during the process. In the figure, the shielding beam loading block is a flexible body, while the loading cylinder and loading pad are both rigid bodies. This setup prevents the cylinder and pad from sharing load and deformation during the process, allowing for a better investigation of the strength issues related to the shielding beam loading block. However, since the loading contact surface is a curved surface, changes in the contact angle between the shielding beam loading block and the pad occur during the loading process, resulting in oscillations in the loading force.

### 2.2 Dynamic coupling characteristics and control challenges

Unlike traditional synchronous control scenarios where dual cylinders operate on relatively independent loads, the 50000kN hydraulic support protection beam loading test bench studied in this paper exhibits unique and complex dynamic coupling characteristics, which pose significant challenges to achieving high-precision synchronous control. These characteristics are primarily manifested in the following aspects:

1. Strong Mechanical Coupling and Load Sensitivity: The two loading hydraulic cylinders do not drive their respective loads independently. Instead, they act jointly on a large protection beam with structural flexibility. Consequently, a minor displacement or force variation in one cylinder is directly transmitted to the other through the beam’s deformation and vibration, affecting its state. This strong mechanical coupling effect creates a high degree of dynamic correlation between the two subsystems, making traditional decoupling control methods difficult to apply.
2. Time-Varying and Nonlinear Dynamics: The dynamic characteristics of the system are not constant. During the loading process, the contact points between the loading pads and the protection beam undergo continuous and nonlinear shifts due to the beam’s motion. This change in contact geometry directly leads to real-time variations in the equivalent loading lever arms and system stiffness. Therefore, the controller must deal with a time-varying, position-dependent nonlinear system model, which is a challenge that fixed-parameter controllers struggle to handle effectively.
3. Asymmetric Disturbances and Oscillations: The loading process of the test bench is prone to introducing asymmetric external disturbances, such as minor force differences caused by structural asymmetries or variations in hydraulic fluid flow. Under the effect of strong mechanical coupling, these disturbances can be amplified and can easily excite the structural modes of the system, leading to oscillations that are difficult to suppress.

In summary, the synchronous control problem of this test bench is not merely a simple position tracking task, but a complex control problem that requires achieving dynamic force balance and high-precision synchronization under conditions of strong coupling, time-varying nonlinearities, and asymmetric disturbances. This is the fundamental reason for adopting the RBF-PID adaptive control strategy in this study, as it can learn and compensate for these complex and difficult-to-model dynamic characteristics online.

## 3 Multi-domain modeling of cover beam loading system

**Fig 4:**
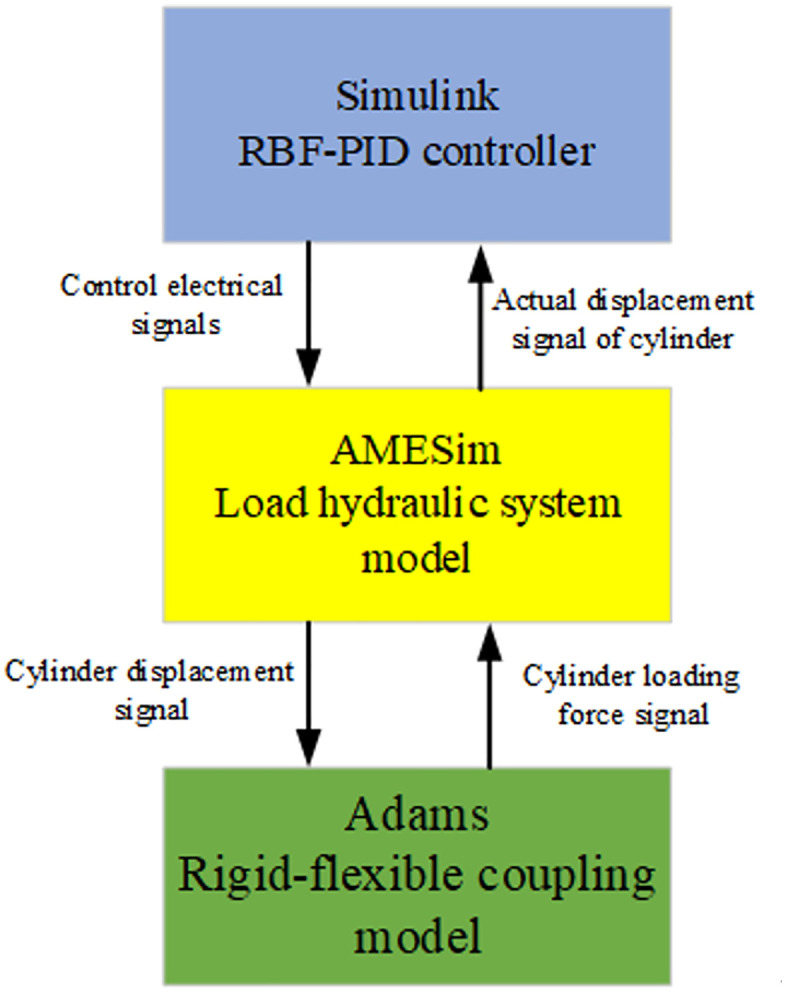
Logic diagram of three-platform joint simulation.

In this simulation experiment, a joint simulation is carried out using the Simulink, AMESim, and Adams platforms [16]. The RBF-PID controller is built in the MATLAB-Simulink module to process signals and transmit control signals to the hydraulic system, accomplishing control over the entire system. The hydraulic system model is constructed in AMESim to receive control signals, control the servo valve, and complete the action of the hydraulic cylinder. The hydraulic cylinder’s driving signals are then transmitted to the 3D model for loading. Lastly, Adams is employed to create a rigid-flexible coupling model of the hydraulic support and the hydraulic support test bench to complete the loading action. The signal transmission process is shown in Fig 4.

### 3.1 Hydraulic power system model construction

**Fig 5:**
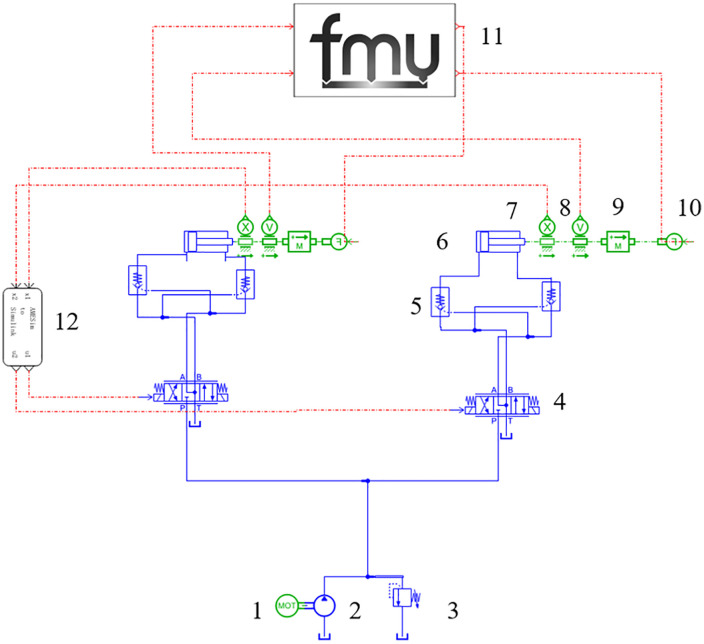
AMESim hydraulic system model. 1-Motor;2-Hydraulic Pump;3-Safety Valve;4-Directional Control Valve;5-Hydraulic Bidirectional Lock;6-Hydraulic Cylinder;7-Displacement Sensor;8- Speed Sensor;9-Equivalent Mass Block;10-Force Conversion Module;11- Adams Mechanical Module;12- Simulink Control Module.

Based on the hydraulic system schematic, the hydraulic system is constructed in AMESim [17–20], as shown in Fig 5. The hydraulic pump station provides oil and pressure to the two loading cylinders, while the solenoid directional valve controls the initiation of the loading action. A hydraulic lock, composed of two fluid-controlled check valves, ensures the stability of the loading device and prevents oil leakage [21–23].

In Fig 5, the FMU module represents the Adams dynamic model module, which receives the displacement signal of the hydraulic cylinder. In the Adams model, the dynamic model input and output are established through the GSE dynamic equation editor. The velocity signal obtained by the dynamic model is used to drive the cylinder model, and its loading force is edited as the output of the module. The AMESim model acquires the load force signal output from this module and feeds it back to the cylinder, thus forming a closed-loop information transfer between the AMESim and Adams models.

The left side of the figure represents the Simulink module, which receives the hydraulic cylinder’s displacement signal. The signal transfer is completed through interaction between the Simulink S-Function and the AMESim model (as shown in Fig 5). The controller calculates the displacement signal, and the electrical signal is output as the module output. AMESim then receives this electrical signal to control the position of the electro-hydraulic proportional servo valve’s orifice. This process forms a closed-loop information transfer between the Simulink and AMESim models. Table 1 shows the parameters of the hydraulic system.

**Table 1:** Hydraulic system parameters.

Parameter content	Numerical value
Hydraulic cylinder diameter	450mm
Piston rod diameter	320mm
Cylinder stroke	1.5m
Mass block	100 kg
Motor speed	1 000r/min
Pump Displacement	4.77ml/r
Proportional valve current	40mA

### 3.2 Control system model construction

#### 3.2.1 Fuzzy PID controller.

The fuzzy PID control algorithm is also one of the commonly used control methods in practical engineering. The fuzzy algorithm determines the correction values for the PID parameters by fuzzifying the input and feedback error values and error change rates, followed by fuzzy inference and defuzzification processes. Fuzzy controllers can control nonlinear systems and exhibit high robustness.

Seven fuzzy concepts are established for input and output signals, namely: negative large (NB), negative medium (NM), negative small (NS), zero (Z), positive small (PS), positive medium (PM), and positive large (PB). The domain of the error e is set to [−1, 1], the domain of ΔKp is set to [−5, 5], the domain of ΔKi is set to [−0.01, 0.01], and the domain of ΔKd is set to [−0.01, 0.01]. The fuzzy rules are imported into the Simulink fuzzy editor, where the input membership function is defined as Gaussian and the output membership function as triangular. The input and output fuzzy rule tables (as shown in Table 2) are edited to complete the setting of fuzzy PID controller rules.

**Table 2:** Fuzzy rule table of fuzzy PID controller.

* ec* *e*	NB	NM	NS	Z	PS	PM	PB
NB	PB NB PS	PB NB NS	PM NM NB	PM NM NB	PS NS NB	Z Z NM	Z Z PS
NM	PB NB PS	PB NB NS	PM NM NB	PS NS NM	PS NS NM	Z Z NS	NS Z Z
NS	PM NB Z	PM NM NS	PM NS NM	PS NS NM	Z Z NS	NS PS NS	NS PS Z
Z	PM NM Z	PM NM NS	PS NS NS	Z Z NS	NS PS NS	NM PM NS	NM PM Z
PS	PS NM Z	PS NS Z	Z Z Z	NS PS Z	NS PS Z	NM PM Z	NM PB Z
PM	PS Z PB	Z Z NS	NS PS PS	NM PS PS	NM PM PS	NM PB PS	NB PB PB
PB	Z Z PB	Z Z PM	NM PS PM	NM PM PM	NM PM PS	NB PB PS	NB PB PB

**Fig 6:**
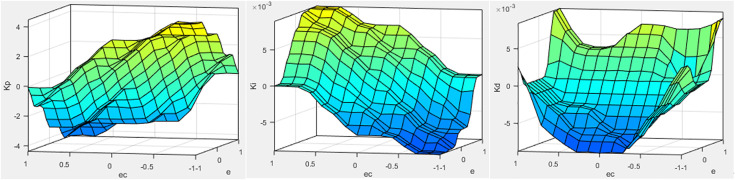
ΔKp ΔKi ΔKd output surface.

**Fig 7:**
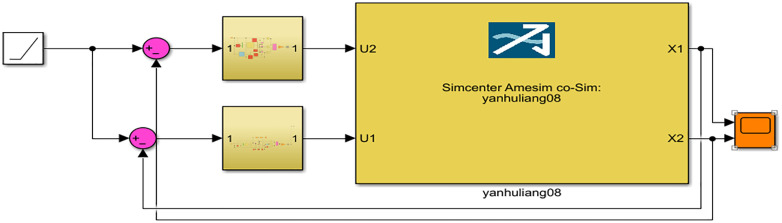
Fuzzy PID parallel control system.

**Fig 8:**
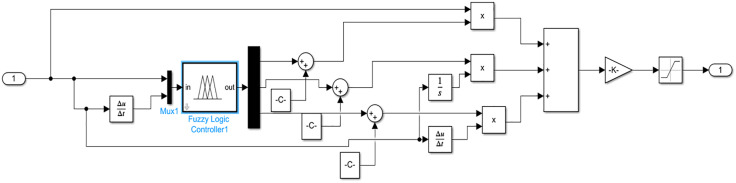
Fuzzy PID control module.

In Simulink, the error and error change rate between the target displacement and the actual displacement of the loading cylinder are used as input variables (Fig 6). The fuzzy rules adjust the PID controller parameters, and the fuzzy PID controller module is constructed in Simulink, as shown in Fig 7. The control system employs an S-function to build the fuzzy PID controller, as illustrated in Fig 8.

#### 3.2.2 RBF-PID controller.

**Fig 9:**
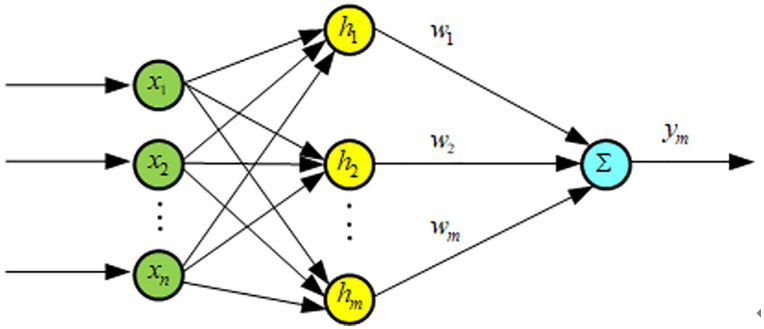
Schematic diagram of RBF neural network.

The Radial Basis Function (RBF) simulates the neural network structure in the human brain, characterized by local adjustments and overlapping receptive fields. It can approximate any continuous function with arbitrary precision. The RBF network is a three-layer feedforward neural network, as shown in Fig 9. The mapping from the input layer to the output layer is nonlinear and represents a local approximation of the neural network. This structure can significantly accelerate learning speed and avoid issues of local minima, making it well-suited for real-time control requirements [24].

The input $x_{n}$ for the network in the RBF network is [25]


$$x={\left[x_{1}\;\;\;x_{2}\;\;\;\cdots \;\;\;x_{n}\right]}^{T}$$ (1)

$h_{j}$ is the Gaussian basis function is the output of the jth neuron of the hidden layer, i.e.,


$$h_{j}(x)=\exp(-\frac{{\|x-c_{j}\|}^{2}}{2{b_{j}}^{2}})\;\;\;\;\;j=1,2,\cdots ,m$$ (2)

where c is the central vector of the hidden layer neurons


$$c=[c_{ij}]=\left[\begin{array}{ccc} {}*20cc_{11} & \cdots & c_{1m}\\ \vdots & & \vdots \\ c_{n1} & \cdots & c_{nm} \end{array}\right]$$ (3)

$$c_{j}=\left[c_{1j}\;\;\;c_{2j}\;\;\;\;\cdots \;\;\;c_{nj}\right]$$ (4)

where b is the width of the Gaussian basis function of the neurons in the hidden layer


$$b={\left[b_{1}\;\;\;b_{2}\;\;\;\cdots \;\;\;\;b_{m}\right]}^{T}$$ (5)

The weights W of the RBF network output are


$$W={\left[w_{1}\;\;\;w_{2}\;\;\;\;\cdots \;\;\;w_{m}\right]}^{T}$$ (6)

The final output of the RBF network is


$$y_{m}\left(t\right)=w_{1}h_{1}+w_{2}h_{2}+\cdots +w_{m}h_{m}$$ (7)

The PID controller is widely used in industrial models due to its simplicity of operation, ease of implementation, and high reliability. However, with technological advancements, its timeliness, lag, and nonlinear issues are becoming increasingly difficult to resolve. By incorporating RBF neural networks to adjust the parameters of the PID controller, its adaptability and robustness can be improved.

The input to the RBF-PID controller is the error value e between the actual displacement and the target displacement with the error rate of change ec.


$$e(k)=y_{d}(k)-y(k)$$ (8)

ec(k)=e(k)−e(k−1) (9)

Output value of the controller u(k):


u(k)=u(k−1)+Δu(k) (10)

where: Δu(k) is the increment of controller output


$$\begin{array}{c} \Delta u(k)=K_{p}[e(k)-e(k-1)]+K_{i}e(k)+\\ K_{d}[e(k)-2e(k-1)+e(k-2)] \end{array}$$ (11)

The performance indicators of this control system rectification index are:


$$E(k)=\frac{\text{1}}{\text{2}}e(k{)}^{2}$$ (12)

To calculate the output weight of the RBF neural network, as well as the center vector and width of the Gaussian basis function, the learning rate η and momentum factor α of the system must be determined. A value of η = 0.25 and α = 0.05 are taken for calculation. The iterative calculation process is as follows [24–25]:


$$\left\{ \;\;\;\begin{array}{c} w_{j}(k)=w_{j}(k-1)+\eta [y(k)-y_{m}(k)]h_{j}\\ +\alpha [w_{j}(k-1)-w_{j}(k-2)]\\ b_{j}(k)=b_{j}(k-1)+\eta [y(k)-y_{m}(k)]w_{j}h_{j}\\ \frac{{\left\|X-C_{j}\right\|}^{2}}{b^{3}j}+\alpha [b_{j}(k-1)-b_{j}(k-2)]\\ c_{ji}(k)=c_{ji}(k-1)+\eta [y(k)-y_{m}(k)]w_{j}h_{j}\\ \frac{x_{i}-c_{ji}}{b^{2}j}+\alpha [c_{ji}(k-1)-c_{ji}(k-1)] \end{array}\right.$$ (13)

The algorithm for determining the rate of change of Kp, Ki, and Kd for the PID controller is obtained by optimizing the parameters of the RBF neural network. The calculation process is shown in Equation 14.


$$\left\{ \;\;\;\begin{array}{c} \Delta K_{p}(k)=\eta_{p}e(k)\frac{\partial y(k)}{\partial u(k)}[e(k)-e(k-1)nonumber\\ \Delta K_{i}(k)=\eta_{i}e(k)\frac{\partial y(k)}{\partial u(k)}e(k)\\ \Delta K_{d}(k)=\eta_{d}e(k)\frac{\partial y(k)}{\partial u(k)}[e(k)-2e(k-1)\\ +e(k-2)] \end{array}\right.$$ (14)

where $\eta_{p}$, $\eta_{i}$, $\eta_{d}$ is the learning rate of each parameter of the PID are taken as 0.02, $\frac{\partial y(k)}{\partial u(k)}$ is the input to output Jacobain information, can be expressed as:


$$\frac{\partial y(k)}{\partial u(k)}\approx \frac{\partial y_{m}(k)}{\partial u(k)}=\sum_{j=1}^{m}w_{j}(k)h_{j}(k)\frac{c_{ji}(k)-x_{i}}{b_{j}(k)}$$ (15)

The RBF-PID parameters are shown in Table 3.

**Table 3:** RBF-PID Parameters.

Parameter Name	Value
Learning Rate *η*	0.25
Momentum Factor α	0.05
Centers of Radial Basis Functions *c*_*i1*_	30
Standard Deviation of Radial Basis Functions *b*_*i1*_	40
Weights of Radial Basis Functions *w*_*1*_	10
*Kpid*	0.3, 0.01, 0.03
Learning Rate *ηpid*	0.02, 0.02, 0.02

**Fig 10:**
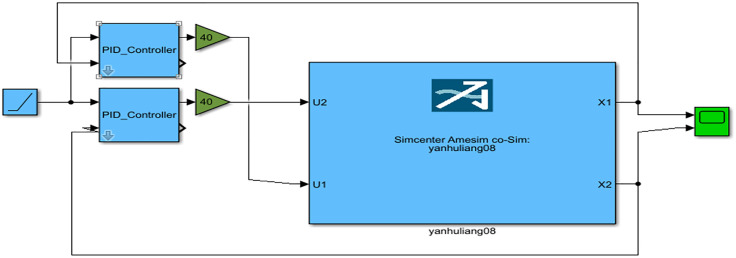
RBF-PID parallel control system.

**Fig 11:**
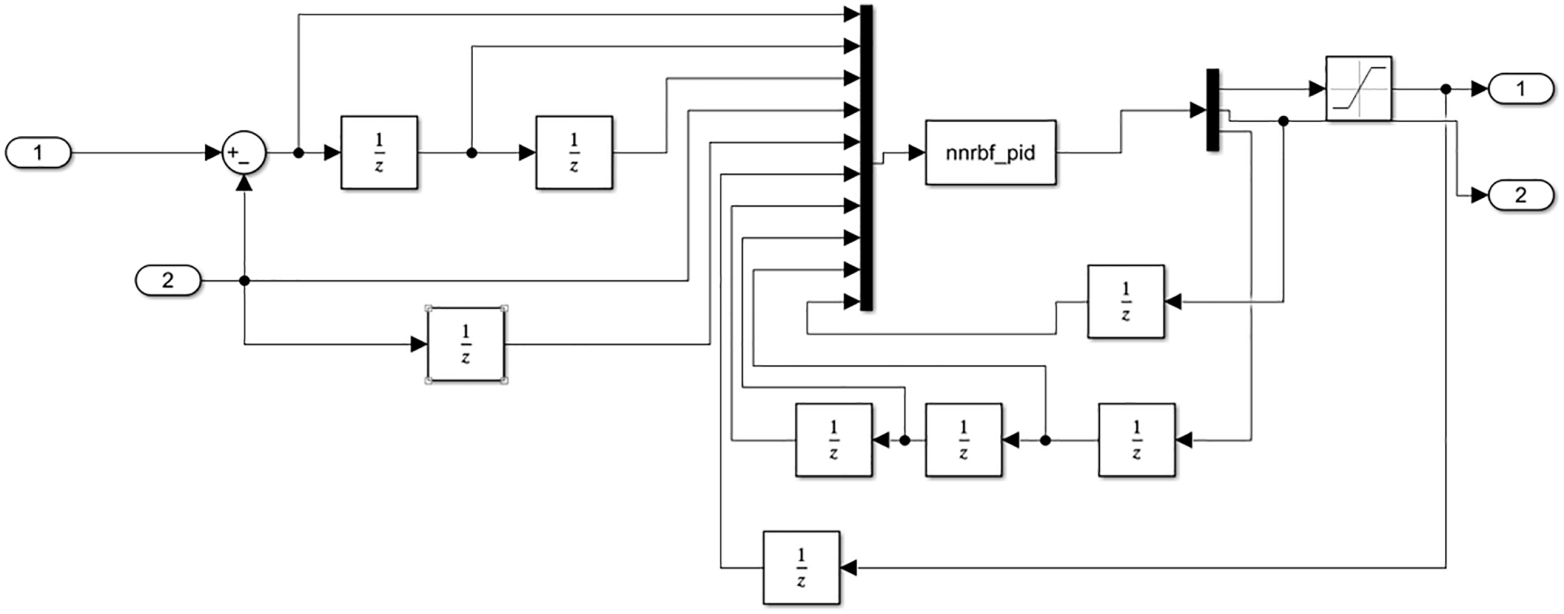
RBF-PID control module.

In Simulink, parallel control strategy is used to achieve position synchronization control of the dual hydraulic cylinders. The control module is shown in the figure below. The PID-Controller module in Fig 10 represents the RBF-PID controller, and the controller module is shown in Fig 11.

### 3.3 Stability analysis of the BRF-PID Controller

To theoretically guarantee the stability of the proposed RBF-PID adaptive controller, a stability analysis based on Lyapunov’s second method is presented in this section. We first establish a mathematical model of the valve-controlled cylinder system that represents the core dynamics of the system.

#### 3.3.1 System error dynamics model.

A typical electro-hydraulic servo valve-controlled cylinder system can be simplified and described by a third-order differential equation:


$$\frac{V_{t}}{4\beta_{e}}\dddot{y}+\frac{A^{2}}{m}\dot{y}+\frac{K_{ce}}{A}\dddot{y}=u(t)-\frac{F_{L}}{A}$$

where *y* is the piston displacement, *u(t)* is the control input (related to the valve spool displacement), and other parameters represent system properties.

This model can be rewritten in the state-space form $\dot{X}=f(X)+g(X)u(t)+d(t)$,where f(X) and g(X) are nonlinear functions containing system parameters, and *d(t)* is a lumped disturbance.

The control objective is to make the system output *y(t)* track a desired trajectory *y*_*d*_*(t)*. The tracking error is defined as *e(t)*=*y*_*d*_*(t)*-*y(t)*. For stability analysis, we construct a sliding variable *s(t)*:


$$s(t)=ce(t)+\dot{e}(t)$$

Where *c* is a positive constant. When *s(t)* →0, the tracking error *e(t)* also asymptotically converges to zero.

The error dynamics can be derived as:


$$\dot{s}(t)=\phi (X)-\gamma u(t)-\dot{d}(t)$$

Where ϕ(X) is a complex nonlinear function, γ is the control gain, $\dot{d}(t)$ is the equivalent lumped disturbance.

We use an RBF neural network to approximate the uncertain function $\phi (X):\phi (X)=W^{*T}h(X)+\varepsilon (X)$, where W^*^ is the ideal weight vector and ε(X) is the bounded approximation error.

#### 3.3.2 RBF adaptive control law and stability proof.

We design the following RBF adaptive control law:


$$u(t)=\frac{\text{1}}{\gamma }({\hat{W}}^{T}h(X)+k_{s}s(t))$$

Where $\hat{W}$ is the estimate of $W^{*}$, and *k*_*s*_ is a positive feedback gain.

The weight update law is designed as”


$$\dot{\hat{W}}(t)=\Gamma h(X)s(t)$$

Where Γ is a positive definite learning rate matrix.

To verify stability, we choose the following Lyapunov candidate function:


$$V(t)=\frac{\text{1}}{\text{2}}s^{2}(t)+\frac{\text{1}}{\text{2}}tr({\tilde{W}}^{T}\Gamma^{-1}\tilde{W})$$

Where $\tilde{W}=\hat{W}-W^{*}$ is the weight estimation error.

Taking the time derivative of V(t) and substituting the error dynamics (L), control law, and adaptive law, we obtain after simplification:


$$\dot{V}(t)=-k_{s}s^{2}(t)+s(t)(\varepsilon -\dot{d}(t))$$

From Equation P, it can be seen that by choosing a sufficiently large feedback gain *ks* such that the term -*k*_*s*_*s*^*2*^*(t)* dominates the term $s(t)(\varepsilon -\dot{d}(t))$, it can be guaranteed that $\dot{V}(t)\leq 0$. This proves that the system is Uniformly Ultimately Bounded (UUB), meaning all error signals will eventually converge to and remain within a small neighborhood of the origin.

The design philosophy of the RBF-PID controller proposed in this study is consistent with the stability analysis above. The RBF network is used to approximate and compensate for system nonlinearities and uncertainties, while the PID component provides robust feedback control. Through careful tuning of controller parameters in simulation and experiments, we have ensured the stability and convergence of the control system in practical operation, and the results are consistent with the conclusions of this theoretical analysis.

## 4 Simulation experiments

According to the design requirements, the maximum pressure the protective beam loading cylinder can provide is 375 tons, so the maximum loading force of the cylinder should be 375 tons. Under the adjusted model posture in Adams, when the cylinder loading force reaches the rated 375 tons, its displacement needs to be 27.5 mm. According to the oil pump flow rate calculated by the AMESim hydraulic system, the speed of the cylinder is 0.5 mm/s. The simulation time is defined as 100s, and the theoretical displacement curve of the cylinder for 100s is used as the input of the controller in Simulink. The cylinder displacement obtained through joint simulation of Adams, AMESim, and Simulink controllers is used as the output. During the simulation process, the controller approximates the displacement in the simulation to the theoretical displacement, and promptly handles oscillations when they occur, thereby achieving the control objectives.

### 4.1 Loading cylinder displacement curve analysis

**Fig 12:**
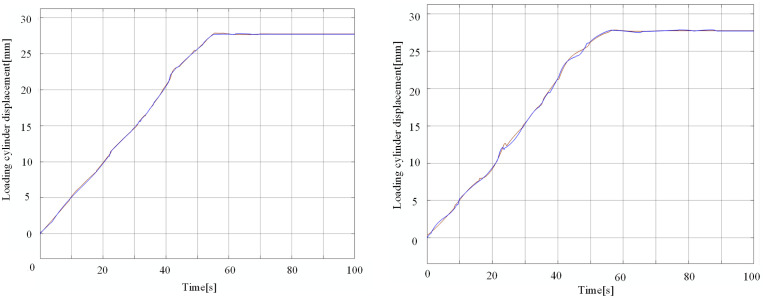
Double-cylinder loading displacement curve. a. RBF-PID control loading displacement curve. b. Fuzzy PID control loading displacement curve.

Through the joint simulation of the three platforms, Simulink draws the displacement curves of the two loading cylinders under the fuzzy PID control and RBF-PID control, as shown in the figure below (Fig 12).

Under the RBF-PID control and fuzzy PID control, the displacement curves both reach the target position at 55.42s and 56.52s, respectively. The displacement curves exhibit oscillations at 23s and 41s during the loading process. The synchronization error of the dual-cylinder under both control methods is shown in the figure below.

A detailed analysis of the displacement curves during the dynamic loading process reveals that challenges to synchronous performance are concentrated at two critical junctures: t ≈ 23s and t ≈ 41s. Our rigid-flexible coupling simulation model allows for the precise observation that a contact dynamics event consistently occurs when the cylinder loading displacement reaches the specific positions of 12–13 mm and 22–23 mm. At these moments, the system’s stiffness and dynamic characteristics undergo a nonlinear change due to an abrupt shift in the contact state between the loading pad and the protection beam.

**Fig 13:**
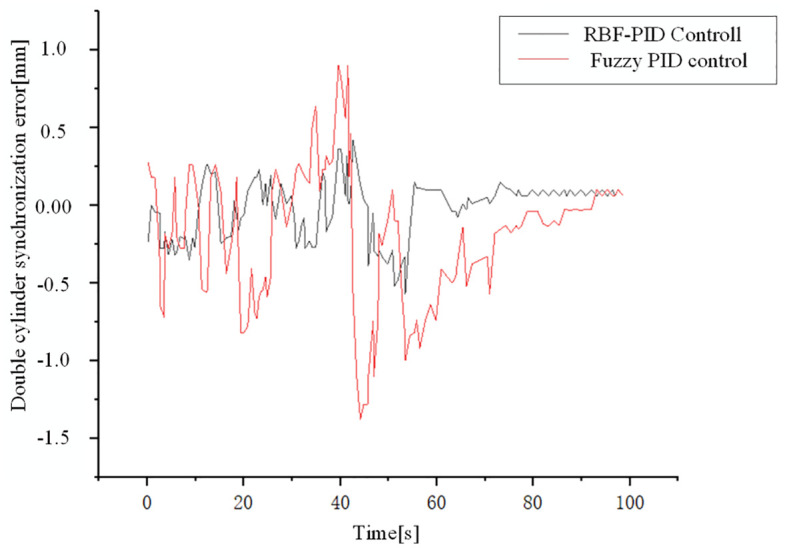
Double cylinder synchronization error of two loading schemes.

Fig 13 contrasts the dual-cylinder synchronization errors obtained with the RBF-PID control and Fuzzy PID control strategies. The simulation results indicate that due to the dynamic changes in the contact position between the loading pad and the loading device throughout the loading process, which leads to complex system dynamic coupling, synchronization errors exhibit oscillations under both control strategies.The RBF-PID control scheme demonstrates superior performance in addressing such dynamic coupling-induced deviations:

Maximum Synchronization Error: The RBF-PID control significantly reduces the maximum synchronization error of the dual-cylinders from 1.3 mm to 0.5 mm.

Oscillation Damping and Convergence Performance: When facing oscillations caused by dynamic contact variations, the synchronization performance under RBF-PID control is notably more stable. The synchronization error converges to zero at 76s, which is approximately 19% faster than the convergence time of 94s observed with Fuzzy PID control. This suggests that the RBF-PID controller can more effectively suppress oscillations during dynamic processes and accelerate the system’s resynchronization.

These findings highlight that the RBF-PID control strategy effectively enhances the precision and robustness of synchronous loading under complex dynamic loading conditions.

### 4.2 Cover beam loading block cloud analysis

The stress contour maps of the two shield beam loading blocks were obtained through post-processing of dynamic simulation under RBF-PID control in Adams.

**Fig 14:**
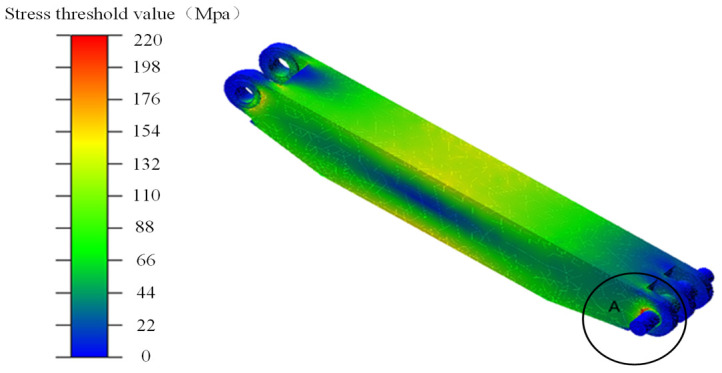
Stress nephogram of shield beam No. 1 loading block.

To facilitate the observation of the overall stress distribution of the shield beam loading blocks, the stress contour map of the shield beam was obtained by taking the stress threshold as 220 MPa according to the simulation results. For the first loading block, most of the stress values are below 220 MPa, as shown in Fig 14. The area with concentrated stress and maximum deformation is located at the pin hole area (position A) in the figure, and the maximum stress value is 298.718 MPa.

**Fig 15:**
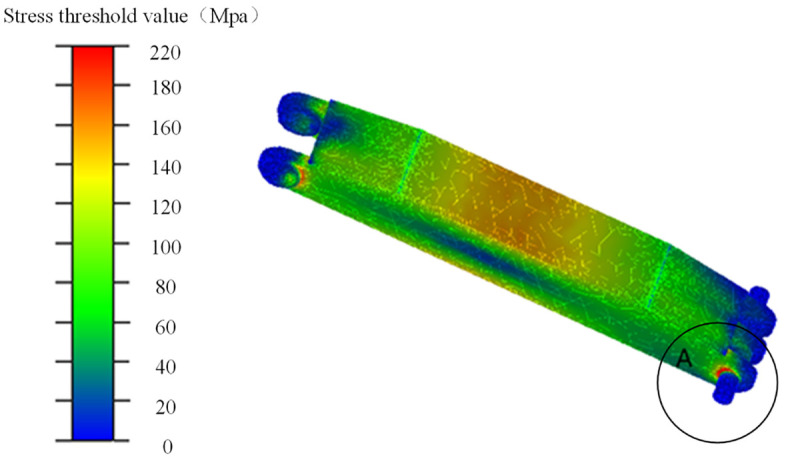
Stress nephogram of shield beam No. 2 loading block.

The stress cloud diagram of the shield beam loading block 2 is shown in Fig 21. As can be seen from Fig 15, most of the stress values of the loading block are below 220 MPa. Similarly, the stress value is the highest in the area around the pinhole (position A), with a maximum value of 322.885 MPa.

The results of synchronized loading with the RBF-PID control strategy show that the flexible loading blocks of both shields have the same stress distribution and reach the maximum stress point at the same location.

## 5 Experimental verification

**Fig 16:**
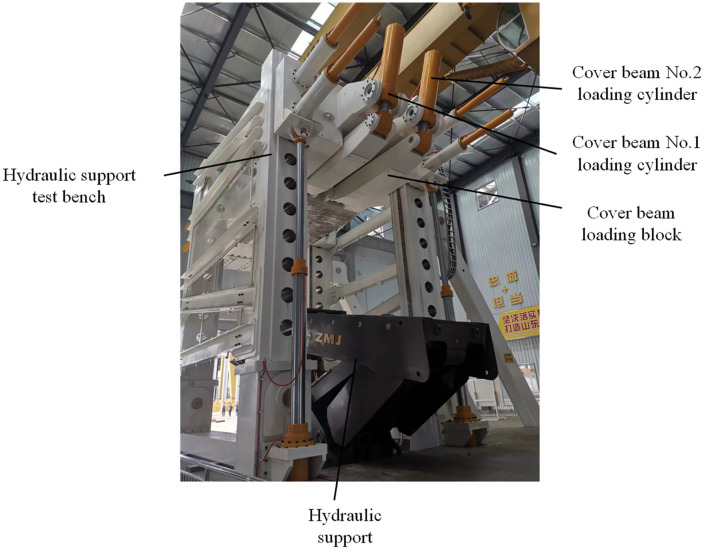
Hydraulic support and test bench.

The 50000KN hydraulic support test platform developed by the project team was subjected to type tests on a 10-meter large support prototype at Donghua Heavy Industry Co., Ltd., completing more than 80,000 compression tests in total, as shown in Fig 16.

The present experiment is a collaboration between the latest 50000KN hydraulic bracket test platform and the Zhengzhou Coal Mining Machinery Group’s ZY29000/45/100D bracket model, with a focus on the strength loading of the high-low position canopy beam.

**Fig 17:**
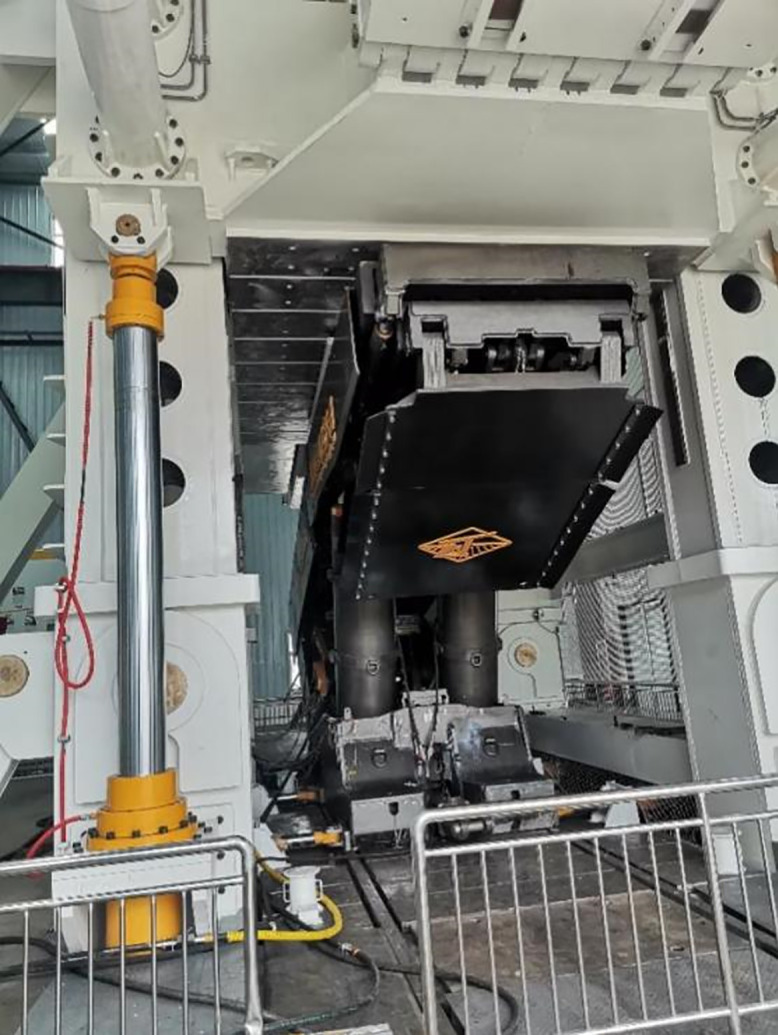
Dual machine attitude adjustment.

**Fig 18:**
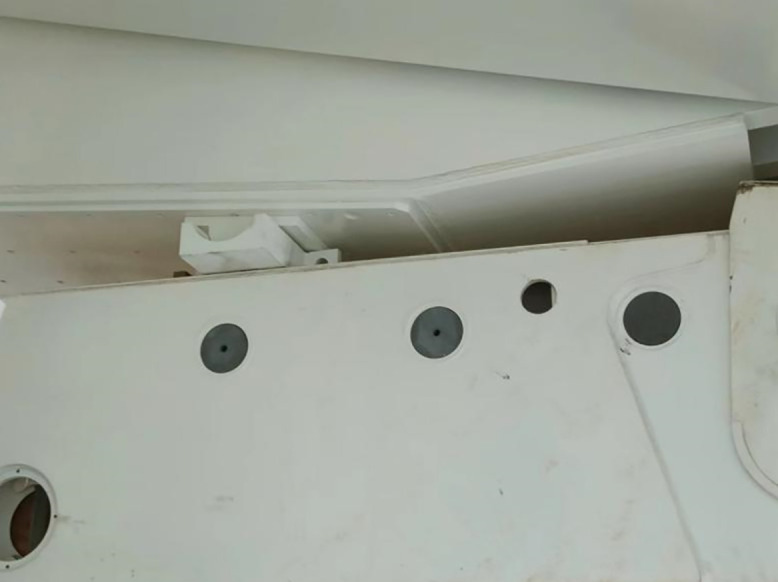
Shield beam loading pad.

**Fig 19:**
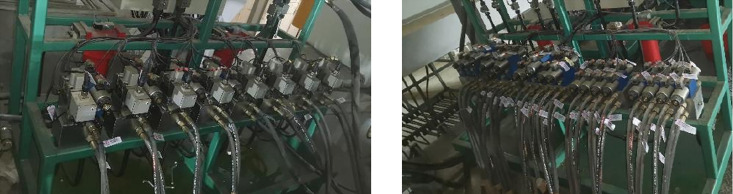
Hydraulic support test bench valve assembly.

**Fig 20:**
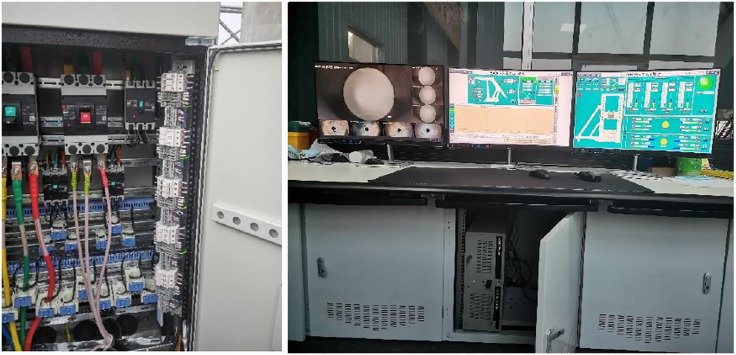
Measurement and control system.

The loading of the canopy beam focuses on the low position loading condition, and it is necessary to adjust the hydraulic bracket to the low position state in cooperation with the test platform. The state of the two machines after adjustment, the position of the canopy beam loading pad, and the control console are shown in the figure below (Fig 17–20).

**Fig 21:**
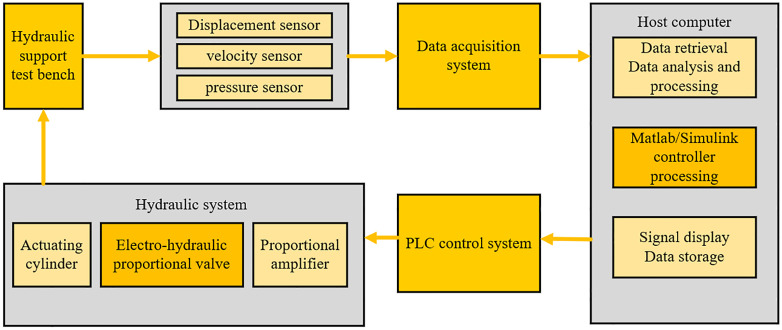
Experimental logic diagram.

The hydraulic support test bench measurement and control system can achieve automatic control and data acquisition of the system. To fulfill its function, the lower computer of the measurement and control system adopts Siemens S7-150 PLC, distributed I/O system, Advantech industrial computer, touchscreen, control cabinet, etc. The control logic diagram is shown in Fig 21.

Based on the optimal parameters identified through extensive simulation, we proceeded with the experimental tuning of the RBF-PID controller. Recognizing that the actual hydraulic system exhibits nonlinearities, sensor noise, and dynamic uncertainties not fully captured in the simulation model, we adopted an iterative approach to refine the controller parameters. The RBF learning rate was slightly reduced from 0.25 to 0.18 to enhance stability in the presence of experimental noise; The momentum factor was kept at 0.05 for its effectiveness in smoothing weight updates; The RBF centers were retained at 30, but their distribution was slightly adjusted based on observed error ranges. The standard deviations were finely tuned to 35 to better capture the nuanced dynamics;The initial PID gains were adjusted based on real-time response: Kp was increased from 0.3 to 0.45, Ki from 0.01 to 0.015, and Kd was slightly decreased from 0.03 to 0.025 to improve responsiveness while maintaining stability; The learning rate for PID parameters was kept at 0.02, as it provided adequate adaptation without introducing instability.

The final set of experimental parameters that yielded the best performance in terms of synchronization accuracy and robustness against loading oscillations are listed in Table 4. These parameters were validated through multiple experimental runs, confirming their effectiveness in the practical operation of the hydraulic support test bench.

**Table 4:** RBF-PID parameters in experiment.

Parameter Name	Value
Learning Rate *η*	0.18
Momentum Factor α	0.05
Centers of Radial Basis Functions *c*_*i1*_	30
Standard Deviation of Radial Basis Functions *b*_*i1*_	35
Weights of Radial Basis Functions *w*_*1*_	10
*Kpid*	0.45, 0.015, 0.025
Learning Rate *ηpid*	0.02, 0.02, 0.02

Based on the experimental plan and the output flow rate of the pump station, calculate the theoretical curve of the cylinder displacement as the input to the control system. The controller drives and controls the loading cylinder of the canopy beam based on the input theoretical curve.

**Fig 22:**
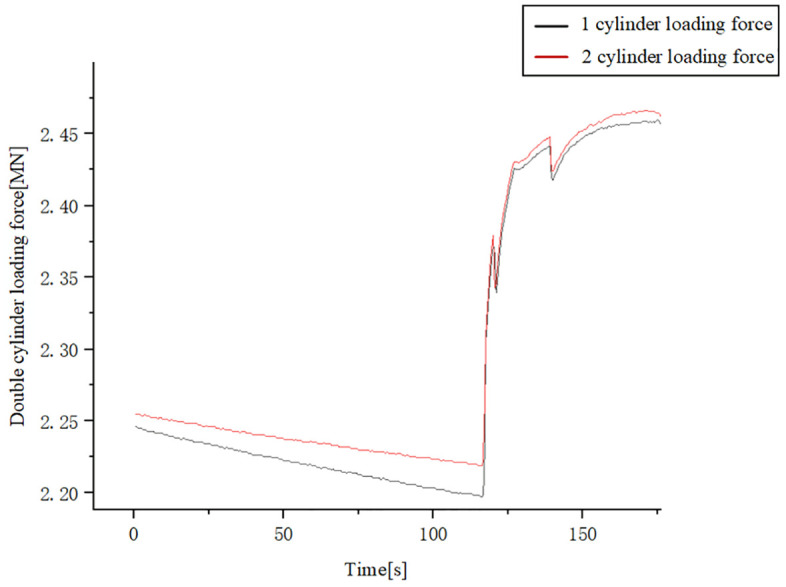
Experimental double-cylinder loading force curve.

Extracting the experimental results at 175s with a data extraction interval of 0.5s, the loading action started at 115s. At the end of loading, the loading force of cylinder 1 reached 246.27t, and the loading force of cylinder 2 reached 245.65t. After loading commenced at 115s, the contact position of the loading pad with the loading device dynamically adjusted with changes in the loading force. This dynamic adjustment in contact led to complex system dynamic coupling, subsequently inducing oscillations in the synchronization error. The degree of impact of this factor on the system can be directly quantified by the loading force fluctuations. As shown in Fig 22, during the loading phase, the experimentally measured loading force curve exhibits sharp, high-frequency fluctuations, with a fluctuation range of up to approximately 0.05 MN.

**Fig 23:**
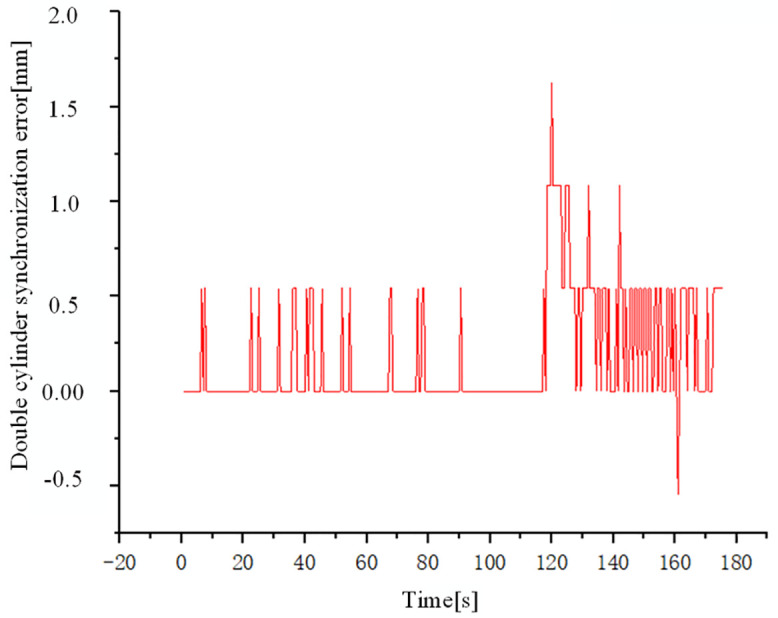
Double cylinder synchronous error curve.

**Fig 24:**
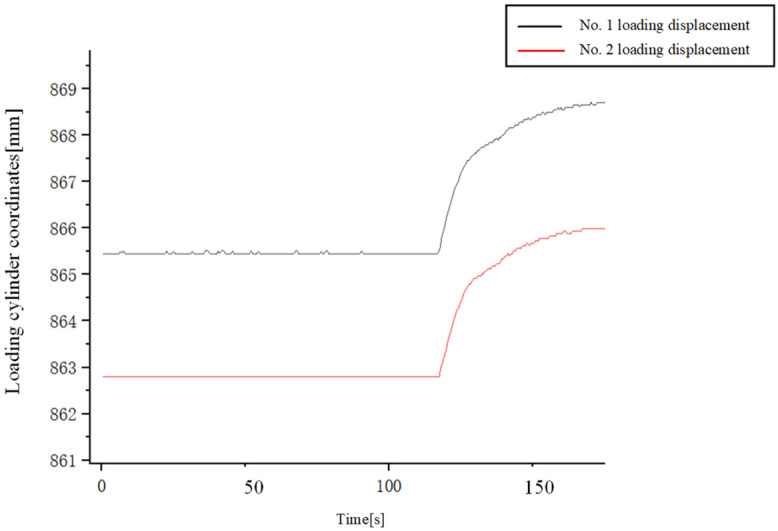
Displacement curve of experimental double cylinder.

Fig 23 illustrates the evolution of dual-cylinder synchronization error during the experiment. In the initial state of the experiment, there was a certain position error between the double cylinders. After loading commenced at 115s, the loading displacement of the double cylinders gradually increased. Throughout this process, the contact position of the loading pad with the loading device dynamically adjusted with changes in the loading force. A combined analysis of the experimental data allows for the precise localization of the point where synchronous performance was most significantly challenged. As shown in Fig 23, the most severe oscillation during the experiment occurred at t ≈ 120s, where the synchronization error reached its peak of 1.65 mm. Mapping this moment back to the displacement curves in Fig 24, it is determined that this oscillation event took place when the two loading hydraulic cylinders reached the specific positions of approximately 867.2 mm and 864.2 mm, respectively. This significant oscillation, occurring at these fixed positions, indicates a critical transition in the dynamic characteristics of the loading process at this displacement point.

This dynamic adjustment in contact led to complex system dynamic coupling, subsequently inducing oscillations in the synchronization error. Experimental results demonstrate that the RBF-PID control algorithm effectively suppresses these oscillations when confronted with synchronization errors caused by dynamic contact variations: the measured maximum synchronization error was 1.65 mm. Furthermore, these experimental findings are consistent with our simulation analysis, confirming the effectiveness and robustness of the RBF-PID control strategy in maintaining high-precision synchronous control, even under complex working conditions involving dynamic contact changes.

## 6 Conclusions

(1) A hydraulic bracket test bench control system for shield loading was established by combining neural network and fuzzy technology with PID controller using multi-domain modeling and simulation technology.
(2) RBF-PID controller and fuzzy PID controller were designed based on simulink to control the dual-cylinder loading. The maximum synchronization error of the dual-cylinder under RBF-PID control was reduced by 61% compared to fuzzy PID control, and the speed of resynchronization after oscillation disturbance increased by 34%. The results show that the RBF-PID controller handles loading disturbances faster and has better performance.
(3) The stress distribution of the shield loading block is the same, indicating that the two loading cylinders under the control of the controller make the shield evenly loaded and indicate that the maximum stress point is at the pinhole connection position, providing data basis for the strength research of the shield loading block.
(4) The 50000kN hydraulic support test bench developed by the project team has been successfully put into operation. Several sets of real-machine tests were conducted using the control method proposed in this study. These experiments addressed the challenges encountered during the loading process of canopy beams on the hydraulic support test bench, providing effective engineering design solutions. Furthermore, the control strategies applicable to engineering conditions were validated and optimized, offering new insights and methods for the development of hydraulic support test benches.

## Supporting information

S1 File S1_Data_Pressure.csv. This CSV file contains the time-series data for the pressure measurements of the two loading hydraulic cylinders recorded during the experimental validation. S2_Data_Displacement.csv. This CSV file contains the time-series data for the displacement measurements of the two loading hydraulic cylinders recorded during the experimental validation, which were used to generate Figs 23 and 24.
(RAR)
